# Progress Toward Global Dracunculiasis (Guinea Worm Disease) Eradication, January 2023–June 2024

**DOI:** 10.15585/mmwr.mm7344a1

**Published:** 2024-11-07

**Authors:** Donald R. Hopkins, Adam J. Weiss, Sarah Yerian, Yujing Zhao, Sarah G.H. Sapp, Vitaliano A. Cama

**Affiliations:** ^1^The Carter Center, Atlanta, Georgia; ^2^Division of Parasitic Diseases and Malaria, National Center for Emerging and Zoonotic Infectious Diseases, CDC.

**Summary**

**What is already known about this topic?**

Human cases of dracunculiasis decreased from an estimated 3.5 million in 1986 to 13 in 2022. The circulation of dracunculiasis in dogs since 2012 has complicated eradication efforts.

**What is added by this report?**

Fourteen human cases and 886 animal infections were reported in 2023, and three human cases and 297 animal infections were reported during January–June 2024. As of June 2024, dracunculiasis remained endemic in five countries (Angola, Chad, Ethiopia, Mali, and South Sudan).

**What are the implications for public health practice?**

Program efforts have brought dracunculiasis close to the goal of eradication. However, dog infections and impeded access due to civil unrest and insecurity in Mali threaten the near-term possibility of global eradication.

## Abstract

The effort to eradicate *Dracunculus medinensis*, the etiologic agent of dracunculiasis, or Guinea worm disease, began at CDC in 1980. In 1986, with an estimated 3.5 million global cases in 20 African and Asian countries, the World Health Assembly called for dracunculiasis elimination. The Guinea Worm Eradication Program (GWEP) was established to help countries with endemic dracunculiasis reach this goal. GWEP is led by The Carter Center and supported by partners, including the countries with endemic disease, CDC, UNICEF, and the World Health Organization. Since 2012, infections in dogs, cats, and baboons have posed a new challenge for GWEP, as have ongoing civil unrest and insecurity in some areas. As of June 2024, dracunculiasis remained endemic in five countries (Angola, Chad, Ethiopia, Mali, and South Sudan). Fourteen human cases and 886 animal infections occurred, including 407 dogs in Chad and 248 dogs in Cameroon, reported in 2023, and three human cases and 297 animal infections reported during January–June 2024. Animal infections, primarily in dogs in Cameroon and Chad, and impeded access due to civil unrest and insecurity in Mali, threaten the near-term possibility of global eradication. Nevertheless, countries appear poised to reach zero cases. 

## Introduction

Dracunculiasis (Guinea worm disease), caused by the parasite *Dracunculus medinensis*, is acquired by drinking water containing small crustacean copepods (water fleas) infected with *D. medinensis* larvae (*1*). Recent evidence suggests the parasite might also be transmitted by eating inadequately cooked fish or other aquatic animals (*2*). Approximately 1 year after infection, the worm emerges through the skin (usually on one of the host’s lower limbs), causing pain and disability (*1*). No vaccine or medicine is available to prevent or treat dracunculiasis. Eradication relies on case containment,* tethering of infected dogs, and other interventions to prevent infection, including health education, water filtration, treatment of unsafe water with temephos (an organophosphate larvicide), provision of safe drinking water, adequate cooking of aquatic animals, and safe disposal of fish entrails (*1*–*4*). CDC began worldwide eradication efforts in 1980, and in 1984, was designated by the World Health Organization (WHO) as the technical monitor of the Dracunculiasis Eradication Program (*1*). In 1986, with an estimated 3.5 million human cases^†^ occurring annually in 20 African and Asian countries^§^ (*5*), the World Health Assembly called for dracunculiasis elimination. The Guinea Worm Eradication Program (GWEP),^¶^ led by The Carter Center and supported by partners that include CDC, UNICEF, and WHO, began assisting ministries of health in countries with endemic disease. Since 1986, WHO has certified 200 countries, areas, and territories as dracunculiasis-free. Five countries with ongoing endemic dracunculiasis (Angola, Chad, Ethiopia, Mali, and South Sudan), plus Sudan, which has not yet completed its dossier and follow-up visit, have not been certified by WHO.**

Since 2012, eradication efforts have been challenged by animal infections, mostly in domestic dogs, and especially in Chad (*6*) in a pattern that remains peculiar to that country (*7*), and the confirmation of human and animal dracunculiasis cases in Angola since 2018.^††^ Worms from infected animals were genetically confirmed to be *D. medinensis* (*8*). GWEP has responded to these challenges by developing and implementing novel strategies. This report updates previous reports^§§^ (*3*) and describes progress toward zero cases during January 2023–June 2024.

## Methods

### Country Reports

Each country’s GWEP provided data on *D. medinensis* infections in humans and animals during January 2023–June 2024. Programs receive monthly case reports from supervised volunteers in each village under active surveillance.^¶¶^ Supervisors review the reports of human and animal infections and verify case containment at regional and national levels, where epidemiologic investigation of all human cases and selected animal infections are also analyzed. Specimens requiring laboratory confirmation are sent to CDC. Villages where endemic transmission has ended (i.e., zero human cases or animal infections reported for ≥12 consecutive months) are kept under active surveillance for 2 additional years.

### WHO Certification of Eradication

WHO certifies a country as dracunculiasis-free after adequate nationwide surveillance for ≥3 consecutive years with no indigenous human case or animal infection.*** This activity was reviewed by CDC, deemed not research, and was conducted consistent with applicable federal law and CDC policy.^†††^

## Results

### Human and Animal Cases

During 2023, a total of 14 human cases of Guinea worm disease were identified in Cameroon, Central African Republic, Chad, Mali, and South Sudan, compared with 13 in 2022, representing an 8% increase (Table 1), but a significant decrease when compared with 5,911 cases in 2007 (Supplementary Figure, https://stacks.cdc.gov/view/cdc/168543). The three human cases identified during January–June 2024 represent no change compared with the same period during 2023. Angola, Cameroon, Chad, Ethiopia, Mali, and South Sudan reported 886 animal infections (mostly in dogs) in 2023, a 30% increase from 2022 (Table 2), although a significant decrease when compared with 1,944 dog infections in 2019 (Supplementary Figure, https://stacks.cdc.gov/view/cdc/168543). Overall, Chad reported approximately one half of the world’s remaining *D. medinensis* infections in human and animals, nearly 90% of which were in dogs. During January–June 2024, animal infections declined 45%, from 540 to 297, during the same period in 2023. No change in the number of human cases (three in January–June 2023 and January–June 2024) occurred during this period. Epidemiologic investigations of human dracunculiasis cases identified the probable location of infection in four of 14 cases in 2023 compared with 11 of 13 in 2022.

**TABLE 1:** Reported dracunculiasis human cases and animal infections, surveillance, and status of local interventions in villages with endemic disease, by country — worldwide, 2023

Characteristic	Country						
	Angola	Cameroon	Chad*	Ethiopia	Mali^†^	South Sudan	Total
**Reported human cases**							
No. indigenous	0	1	10	0	1	2	**14**
No. imported	0	0	0	0	0	0	**0**
% Contained^§^ (no./total no.)	NA	100 (1/1)	60 (6/10)^¶^	NA	— (0/1)	— (0/2)	**50 (7/14)**
% Change in indigenous human cases in villages or localities under surveillance (no. in 2022 vs. 2023)	NA (0 vs. 0)	NA (0 vs. 1)	43 (7 vs. 10)	–100 (1 vs. 0)	NA (0 vs. 1)	–60 (5 vs. 2)	**8 (13 vs. 14)**
**Reported animal cases**							
No. indigenous	85	255	497	1	47	1	**886**
No. imported	0	0	0	0	0	0	**0**
% Contained^§^ (no./total no.)	2 (2/85)	87 (221/255)	76 (377/497)	100 (1/1)	74 (35/47)	— (0/1)	**72 (636/886)**
% Change in indigenous animal infections in villages or localities under surveillance (no. in 2022 vs. 2023)	1,114 (7 vs. 85)	811 (28 vs. 255)	–17 (602 vs. 497)	–67 (3 vs. 1)	15 (41 vs. 47)	0 (1 vs. 1)	**30 (682 vs. 886)**
**Villages under active surveillance**							
No. of villages reporting monthly (%)	158 (100)	26 (100)	2,768 (100)	200 (100)	1,965 (100)	2,584 (100)	**7,701 (100)**
No. reporting one or more human case	0	1	6	0	1	2	**10**
No. reporting only imported human cases	0	0	0	0	0	0	**0**
No. reporting indigenous human cases	0	1	6	0	1	2	**10**
No. reporting one or more animal infection	59	15	260	1	22	1	**358**
No. reporting only imported animal infections	0	0	0	0	0	0	**0**
No. reporting indigenous animal infections	59	15	260	1	22	1	**358**
**Status of interventions in villages with endemic human dracunculiasis**							
No. of villages with endemic human dracunculiasis, 2022–2023	0	1	12	1	1	5	**20**
% Reporting monthly (no./total no.)	NA	100 (1/1)	100 (12/12)	100 (1/1)	100 (1/1)	100 (5/5)	**100 (20/20)**
% Filters in all households (no./total no.)	NA	100 (1/1)	75 (9/12)	100 (1/1)	100 (1/1)	100 (5/5)	**85 (17/20)**
% Using temephos (no./total no.)	NA	100 (1/1)	75 (9/12)	100 (1/1)	100 (1/1)	100 (5/5)	**85 (17/20)**
% One or more source of safe water (no./total no.)	NA	100 (1/1)	75 (9/12)	— (0/1)	100 (1/1)	80 (4/5)	**75 (15/20)**
% Provided health education (no./total no.)	NA	100 (1/1)	83 (10/12)	100 (1/1)	100 (1/1)	100 (5/5)	**90 (18/20)**
**Status of interventions in villages with endemic animal dracunculiasis**							
No. of villages with endemic animal dracunculiasis, 2022–2023	62	26	436	3	41	2	**570**
% Reporting monthly (no./total no.)	100 (62/62)	100 (26/26)	100 (436/436)	100 (3/3)	100 (41/41)	100 (2/2)	**100 (570/570)**
% Using temephos (no./total no.)	23 (14/62)	85 (22/26)	88 (383/436)	100 (3/3)	76 (31/41)	100 (2/2)	**80 (455/570)**
% Provided health education (no./total no.)	100 (62/62)	100 (26/26)	92 (401/436)	100 (3/3)	100 (41/41)	100 (2/2)	**94 (535/570)**

**Abbreviations:** GWEP = Guinea Worm Eradication Program; NA = not applicable.

* Participants at the annual Chad GWEP review meeting in November 2014 adopted “1+ case village” as a new description for villages in Chad affected by human cases of Guinea worm disease or dogs infected with Guinea worms and defined it as “a village with one or more indigenous and/or imported cases of Guinea worm infections in humans, dogs, and/or cats in the current calendar year and/or previous year.”

^†^ Civil unrest and insecurity since a coup in 2012 continued to constrain GWEP operations (supervision, surveillance, and interventions) in Gao, Kidal, Mopti, Segou, and Timbuktu Regions.

^§^ Human cases are contained when all of the following criteria are met: 1) infected patients are identified ≤24 hours of worm emergence; 2) patients have not entered any water source since the worm emergence; 3) a village volunteer or health care provider has properly treated the lesion until all detectable worms are fully removed and has educated the patient on how not to contaminate water sources; 4) the containment process is validated by a GWEP supervisor ≤7 days of worm emergence; and 5) all contaminated and potentially contaminated sources of drinking water are treated with temephos. The criteria for defining a contained case of dracunculiasis in a human should also be applied, as appropriate, to define containment for an animal with Guinea worm infection.

^¶^ A total of six human cases were reported from Chad in 2022 and nine in 2023. One human case was reported from the Central African Republic in 2022 and one in 2023. These two human cases might have been acquired in Chad.

**TABLE 2:** Number of reported indigenous human and animal dracunculiasis cases, by country — worldwide, January 2022–June 2024

Characteristic	Country						
	Angola	Cameroon*	Chad^†^	Ethiopia	Mali^§^	South Sudan	Total
**Human cases**							
**No. of cases (% contained)**							
Jan–Dec 2022	0 (—)	0 (—)	7 (43)	1 (100)	0 (—)	5 (60)	**13 (54)**
Jan–Dec 2023	0 (—)	1 (100)	10 (60)	0 (—)	1 (—)	2 (0)	**14 (50)**
% Change, Jan–Dec 2022 to Jan–Dec 2023	NA	NA	43	–100	NA	–60	**8**
Jan–Jun 2023	0 (—)	1 (100)	2 (100)	0 (—)	0 (—)	0 (—)	**3 (100)**
Jan–Jun 2024	0 (—)	0 (—)	1 (0)	0 (—)	0 (—)	2 (50)	**3 (33)**
% Change, Jan–Jun 2023 to Jan–Jun 2024	NA	–100	−50	NA	NA	NA	**0**
**Animal infections^¶^**							
**No. of cases (% contained)**							
Jan–Dec 2022	7 (0)	28 (100)	602 (70)	3 (33)	41 (63)	1 (100)	**682 (70)**
Jan–Dec 2023	85 (2)	255 (87)	497 (76)	1 (100)	47 (74)	1 (0)	**886 (72)**
% Change, Jan–Dec 2022 to Jan–Dec 2023	1,114	811	–17	−67	15	0	**30**
Jan–Jun 2023	81 (2)	229 (87)	221 (76)	0 (—)	9 (78)	0 (—)	**540 (70)**
Jan–Jun 2024	36 (25)	117 (93)	144 (65)	0 (—)	0 (NA)	0 (—)	**297 (71)**
% Change, Jan–Jun 2023 to Jan–Jun 2024	–56	–49	–35	NA	–100	NA	**–45**

**Abbreviation:** NA = not applicable.

* One human case and multiple animal infections detected in areas of Cameroon near the border with Chad might have been infected in Chad. Cameroon has 117 provisional dog infections and eight provisional cat infections, for which specimens are pending laboratory confirmation.

^†^ Chad’s human case counts for January–December 2022 and January–December 2023, each including one human case detected in an area of the Central African Republic.

^§^ Civil unrest and sociopolitical insecurity since a coup in April 2012 continued to constrain program operations in regions with endemic dracunculiasis (Gao, Kidal, Mopti, Segou, and Timbuktu) during January 2021–June 2024.

^¶^ In Chad, primarily dogs and some cats; in Ethiopia, dogs, cats, and baboons; in Mali, dogs and cats; in Angola, dogs; in Cameroon, dogs and cats.

### Analysis of Laboratory Specimens

During January–June 2024, CDC received seven specimens from humans, only one of which was laboratory-confirmed as *D. medinensis*^§§§^ (Table 3), compared with 15 specimens received, with one confirmed, during January–June 2023. No human cases were reported during January–April and November–December 2023. During January–June 2024, CDC received 482 animal specimens, 434 (90%) of which were laboratory-confirmed *D. medinensis*, compared with 114 (87%) of 131 specimens confirmed during January–June 2023.

**TABLE 3:** Characteristics of human and animal specimens received at CDC for laboratory diagnosis of *Dracunculus medinensis* — January 2023–June 2024

Characteristic	Years/Months			
	2024	2023		
	Jan–Jun	Jan–Jun	Jul–Dec	Jan–Dec
**Human specimens**				
Positive specimens, by country of origin, no. of specimens (no. of patients)*				
Cameroon	—**^†^**	—	1 (1)	1 (1)
Central African Republic	—	—	1^§^ (1)	1 (1)
Chad	1 (1)	1 (1)	9 (8)	10 (9)
Mali	—	—	1 (1)	1 (1)
South Sudan	—	—	2 (2)	2 (2)
**Total no. of positive specimens (%)**	**1 (14)**	**1 (7)**	**14 (36)**	**15 (28)**
Negative specimens, by other laboratory identifications, no. (%)*				
Free-living organism^¶^	—	1 (7)	5 (20)	6 (15)
*Onchocerca* sp.	2 (29)	2 (14)	3 (12)	6 (15)
Other parasitic nematode**	1 (14)	4 (29)	1 (4)	5 (13)
Plant material	—	—	3 (12)	3 (8)
Sparganum	1 (14)	3 (21)	11 (44)	14 (35)
Tissue (animal origin)	1 (14)	1 (7)	2 (8)	3 (8)
Unknown origin	1 (14)	3 (21)	—	3 (8)
Total no. of negative specimens* (%)	**6 (86)**	**14 (93)**	**25 (64)**	**39 (72)**
**Total no. of human specimens**	**7**	**15**	**39**	**54**
**Animal specimens**				
Positive specimens, by country and species of origin, no. of specimens (no. of animals)*				
**Angola**				
Dog	50 (50)	32 (32)	41 (41)	73 (73)
**Cameroon**				
Cat	9 (5)	—	7 (6)	7 (6)
Dog	364 (208)	67 (61)	32 (32)	99 (93)
Other animals (not determined)	—	—	1 (1)	1 (1)
**Chad**				
Cat	—	—	1 (1)	1 (1)
Dog	8 (8)	8 (8)	—	8 (8)
**Ethiopia**				
Baboon	1 (1)^††^	—	—	—
Dog	—	—	1 (1)	1 (1)
Other animal (serval or wildcat)	—	1 (1)	—	1 (1)
**Mali**				
Cat	—	—	5 (5)	5 (5)
Dog	—	6 (5)	37 (35)	43 (40)
Other animal (donkey)	—	—	1 (1)	1 (1)
**South Sudan**				
Wildcat	—	—	1 (1)	1 (1)
Other animal (genet or serval)	2 (2)^††^	—	—	—
**Total no. of animal specimens**	**482**	**131**	**142**	**273**
No. of positive specimens* (%)	**434 (90)**	**114 (87)**	**127 (89)**	**241 (88)**
Total no. of negative specimens* (%)	**48 (10)^§§^**	**17 (13)**	**15 (11)**	**32 (12)** ^¶¶^

* Positive specimens were confirmed as *D.* *medinensis*; negative specimens were ruled out as *D. medinensis*.

^†^ Dashes indicate no specimens received.

^§^ This specimen was collected in November 2023 but arrived at CDC in January 2024; it is reported as 2023.

^¶^ Free-living organisms primarily included adult Mermithidae and other worms identified as belonging to nonparasitic taxa.

** Other parasitic nematodes submitted in association with human cases belonging to the filarioidea or ascarididae families.

^††^ Subcutaneous worm not yet emerged extracted from a dead baboon (worms that have not emerged do not meet case definition and are not counted as cases.

^§§^ The 48 negative specimens from animals from 2024 were identified as follows: 26 were other parasitic nematodes (nine diplotriaenidae, four filariidae, three ascarididae, three gnastostomatidae, three physalopteridae, two spirirudidae, and two nematodes); 12 were spargana; two were free-living organisms; three were animal tissues; one was plant tissue; and four were of unknown origin.

^¶¶^ In 2023, the 32 negative specimens were identified as follows: 16 were other parasitic nematodes (eight filaroidea, three *Setaria* sp., three *Hastopiculum* sp., one spiruroidea, and one strongyloidea), six were free-living organisms (five mermithids), one was animal tissue, likely from fish, and two samples were of unknown origin.

### Country Reports

**Angola.** Angola reported 158 communities under active community-based surveillance in 2023 (Table 1). A total of 85 infected dogs were detected in 2023, and 36 during January–June 2024 (Table 2), all in the same province as in previous years. Genetic analysis to date has not linked Angola’s Guinea worms to *D. medinensis* specimens from other countries (E Thiele, PhD, Vassar College, personal communication, September 2024). Angola offers a cash reward equivalent to US$450 for reporting a human or animal infection. This program continues proactively tethering dogs at risk for infection and using temephos in affected areas.

**Cameroon.** Cameroon reported 255 infected animals (248 dogs and seven cats) and one human case in 2023 and 115 confirmed infected dogs and two cats in January–June 2024 (Table 1) in villages <3 miles (<5 km) from the Chad-Cameroon border. These animals were likely infected in Chad because the affected villages included families living on both sides of the border, and dog owners took their dogs to Chad regularly. Cameroon expanded active surveillance to all villages of concern and continued proactive tethering of dogs in the affected area. 

**Chad.** Chad reported 10 human cases in 2023, including three in the same household (and one human case detected in Central African Republic), compared with six cases in 2022; during January–June 2024, one case was reported, compared with two during the same period in 2023. A total of 496 animal infections (407 dogs and 89 cats) were reported in 2023, 18% fewer than the 601 (516 dogs and 85 cats) reported in 2022. During January–June 2024, Chad reported 35% fewer infected animals (144) compared with 219 during January–June 2023. The Carter Center assisted Chad’s GWEP implementation of village-based surveillance for human and animal infections in 2,768 at-risk villages by December 2023 (Table 1). Active surveillance generated 110,784 rumors (any information about a possible case of Guinea worm disease among humans or animals) during January–June 2023, increasing by 51% to 166,996 rumors during January–June 2024.

Chad’s Ministry of Health continues to offer a reward equivalent to US$100 for reporting a confirmed human dracunculiasis case and a US$20-equivalent reward for reporting an animal infection. Evaluations in areas with established active surveillance indicated that 70% and 89% of residents surveyed during 2023 and January–June 2024, respectively, were aware of the rewards.

In 2013, Chad implemented educational campaigns aimed at preventing dog consumption of fish entrails by burying the entrails. Monthly assessments from 2023 and January–June 2024 showed that 43% and 34%, respectively, of households in at-risk communities were burying fish entrails.

Chad’s GWEP began tethering dogs with dracunculiasis-compatible signs in 2014. These efforts have increased over time, and beginning in 2022, all dogs in all villages reporting one or more dog infections during the preceding or current year are tethered. As a result, 81% and 74% of eligible dogs were tethered during 2023 and January–June 2024, respectively.

Water treatment with temephos reached 87% of 279 villages with dog or human infections by December 2023 and 98% of 96 villages by June 2024. In December 2023, 79% of villages reporting dracunculiasis had at least one source of copepod-free drinking water (e.g., from a borehole well). Advocacy efforts during January 2023–June 2024 included the visit of Chad’s minister of health to an area with endemic dracunculiasis in June 2023, and eight provincial governors signed declarations in February and April–May 2024 pledging definitive action to support eradication.

**Ethiopia.** Ethiopia reported one infected dog and no human cases in 2023; no infected human or animal was reported during January–June 2024 (Table 2). Ethiopia’s public health and wildlife authorities, with assistance of The Carter Center, continued trapping and examining baboons through 2024. Active surveillance was conducted in 198 villages and 223 non-village areas, in an area of about 50 x 25 miles (80 x 40 km) in Gog district and part of adjacent Abobo district. In April 2024, one nonemerged worm was confirmed from a dead baboon; however, nonemerged worms do not meet the case definition and therefore are not included in case counts. The reward for reporting human dracunculiasis cases is equivalent to US$360 and US$40 for reporting and tethering infected animals. In 2023, 96% of persons surveyed in active surveillance areas knew of the rewards; during January–June 2024, 93% were aware.

Since April 2018, Ethiopia has supported villager-initiated constant tethering of approximately 1,900 dogs and cats in villages at highest risk to prevent their exposure to water sources in adjacent forests where transmission apparently occurs. The program applies temephos monthly to nearly all water sources known to have been used by humans or infected animals in the at-risk areas of Abobo and Gog districts. Since 2022, GWEP uses remote sensing from Maxar Technologies (https://www.maxar.com) to identify new water sources that need to be treated.

**Mali.** Mali reported one human dracunculiasis case in 2023 and no human cases during January–June 2024, compared with no cases during January 2022–June 2023 (Table 2). In 2023, 47 infected animals were reported, compared with 41 in 2022. Mali reported no animal infections during January–June 2024, compared with nine infected dogs during the same period in 2023. Among the infected animals identified in 2023, 44 were detected in Segou Region and three in adjacent Mopti Region, in areas relatively inaccessible because of civil unrest. Animals from Segou Region apparently became infected in Mopti Region.

In 2023, a total of 1,965 villages in Mali were under active surveillance (Table 1), with cash rewards equivalent to US$340 offered for reporting a human case and US$20 for reporting and tethering an infected animal. In active surveillance areas in 2023, 84% of persons knew about the rewards for reporting an infected person or animal; during January–June 2024, 98% knew about the rewards. Since late 2021 Mali has been proactively tethering dogs during the June–September peak transmission season, and in 2023, tethering was extended to include puppies.

**South Sudan.** South Sudan reported two human cases in 2023, compared with five in 2022 (Table 2). An infected wildcat (genet) was detected for the first time in November 2023. Two human cases and no infected animals were reported during January–June 2024. The high mobility of cattle herders and others in South Sudan poses a challenge to GWEP surveillance and interventions, as does sporadic sociopolitical insecurity. By December 2023, a total of 2,584 villages in South Sudan were under active surveillance (Table 1). The reward for reporting a human dracunculiasis case was increased from the equivalent of US$375 to US$750, and for reporting an infected animal remained at US$375. Surveys in 2023 found that 66% of respondents in areas with endemic dracunculiasis and 21% in at-risk areas were aware of the rewards. The minister of health visited an area with endemic dracunculiasis to advocate for Guinea worm disease eradication in April 2023.

## Discussion

The 14 human cases of dracunculiasis reported in 2023 represented the second-lowest annual number of human cases ever reported and, for the second consecutive year, no cases were reported for 6 months (January–April and November–December). Progress toward Guinea worm disease eradication was reviewed at the 2023 and 2024 annual meetings of GWEP program managers and at unofficial meetings during the 2023 and 2024 World Health Assemblies. Support from local government leaders continues to be important to sustaining and improving dracunculiasis eradication efforts.

### Infections in Animals

During January 2023–June 2024, animal infections were the main challenge to dracunculiasis eradication. Transmission of *D. medinensis* in Chad is hypothesized to result from consumption of inadequately cooked aquatic animals, including fish or other transport hosts or paratenic hosts (an intermediate host in which no parasite development occurs but which serves to maintain the viability of larval stages of a parasite) (*2*). The high environmental contamination by many infected dogs is driving transmission to a few humans and cats, as well as other dogs. Stopping transmission among dogs is now the GWEP’s primary focus. Angola and Cameroon also reported animal dracunculiasis. These countries improved their surveillance efforts in 2022, leading to a significant increase in reported infections in 2023. Dog infections also predominate in Mali, but at a lower level, and remained approximately the same in 2022 and 2023. Ethiopia found only two infected dogs and two infected baboons in 2022–2023. South Sudan’s improved surveillance efforts led to identifying an infected wild feline, a genet, in 2023.

Overall, animal dracunculiasis increased by 30% between 2022 and 2023 but declined by 45% in January–June 2024 when compared with January–June 2023. In Chad, however, animal dracunculiasis declined for the fourth and fifth consecutive years: by 17% from 602 in 2022 to 497 in 2023 and by 35% from 221 during January–June 2023 to 144 during January–June 2024. The challenge of animal infections, which occur in limited geographic areas except in Chad, is being addressed through innovative interventions and research supported by The Carter Center, CDC, and WHO. After being pioneered by Ethiopia in 2018, proactive tethering of dogs in at-risk villages has proven effective and was adopted by GWEPs in Chad in 2020, Mali and Cameroon in 2022, and Angola in 2023. Baboon infections appear to be declining in Ethiopia because of intensive temephos treatments of water sources in the areas of concern.

### Infections in Humans

The detection of three human cases in two districts in Angola during 2018–2023 and three cases during 2019–2023 in one district in Cameroon that borders an area of Chad with endemic disease suggests that the problems in Angola and Cameroon are limited. Detection of two human cases in the Central African Republic during 2022–2023, bordering an area with endemic dog infections in Chad, also highlights the risks for exportation and the need for ongoing active surveillance and implementation of control measures in neighboring countries.

Adequate security is also important to achieving eradication goals, especially in Mali and South Sudan. Mali’s GWEP has worked with ministry of health, regional, and local leaders in a Peace through Health Initiative (*9*), which relies on health services as an entry point for peacebuilding to reduce sociopolitical insecurity. This initiative started in 2020 in one district; and expanded to four districts in 2022. Mali needs peaceful conditions to facilitate implementation of interventions in all six districts with endemic dracunculiasis during its 6-month transmission season. If adequate security is maintained and transmission is not sustained among animals, Ethiopia and South Sudan appear poised to achieve dracunculiasis elimination through strong technical leadership and national political support.

### Limitations

The findings in this report are subject to at least two limitations. First, the GWEP surveillance activities have some known potential shortcomings, including deliberate underreporting, missed cases or infections, and limited accessibility to all areas with endemic disease due to insecurity and civil unrest. Second, accurately determining the extent of dracunculiasis in wildlife is a substantial challenge to GWEP, although most of the remaining foci appear to be driven by infected domestic animals, mainly dogs.

### Implications for Public Health Practice

Eradicating dracunculiasis will have a significant positive societal impact. Benefits accrued to date include millions of persons no longer being at risk for contracting Guinea worm disease, with associated improvements in their health, agricultural productivity, and education (*10*). Program efforts have also led to increased numbers of trained and experienced health officers and thousands of village volunteers. Achieving dracunculiasis eradication would be a monumental accomplishment because it would likely be the second human disease to be eradicated after smallpox and would have been achieved without a vaccine or curative treatment. GWEP is proactively addressing new challenges, including wildlife infections. Joint efforts with partners and multiple research institutions are helping to elucidate the unusual epidemiology of dracunculiasis in the remaining affected countries and to develop new interventions to reach the goal of eradication.

## Footnotes

* Human cases are contained when all of the following criteria are met: 1) infected patients are identified ≤24 hours of worm emergence; 2) patients have not entered any water source since worm emergence; 3) a village volunteer or health care provider has properly treated the lesion until all detectable worms are fully removed and has educated the patient on how not to contaminate water sources; 4) the containment process is validated by a GWEP supervisor ≤7 days of worm emergence; and 5) all contaminated and potentially contaminated sources of drinking water are treated with temephos. The criteria for defining a contained case of dracunculiasis in a human should also be applied, as appropriate, to define containment for an animal with a Guinea worm infection.

† A dracunculiasis case is defined as an infection occurring in a person exhibiting a skin lesion or lesions with emergence of one or more worms that are laboratory-confirmed as *D. medinensis* at CDC. Because *D. medinensis* has a 10–14-month incubation period, each infected person is counted as having an infection only once during a calendar year.

§ Initially 20 countries, but the former country of Sudan officially separated into two countries (South Sudan and Sudan) on July 9, 2011.

¶
https://www.who.int/activities/eradicating-dracunculiasis

**
https://www.who.int/publications/i/item/who-wer9820-205-224

††
https://www.who.int/publications/i/item/who-wer9721-22-225-247

§§
https://www.who.int/publications/i/item/who-wer-9920-249-269

¶¶ Villages under active surveillance are those that have endemic dracunculiasis or are at high risk for importation. Active surveillance involves daily searches of households by village volunteers (supported by their supervisors) for persons or animals with signs of dracunculiasis.

*** An indigenous dracunculiasis human case or animal infection is defined as an infection consisting of a skin lesion or lesions with emergence of one or more Guinea worms in a person or animal who had no history of travel outside their residential locality during the preceding year.

††† 45 C.F.R. part 46.102(l)(2), 21 C.F.R. part 56; 42 U.S.C. Sect. 241(d); 5 U.S.C. Sect. 552a; 44 U.S.C. Sect. 3501 et seq.

§§§ Specimens are laboratory-identified as *D. medinensis* at CDC by morphologic examination under a microscope or DNA sequencing assays. https://www.cdc.gov/dpdx/dxassistance.html
